# The Knoevenagel-Doebner Reaction on 1,2-*O*-(2,2,2-Trichloroethylidene) Derivatives of D-*Gluco*- and D-*Manno*- *furanose* †

**DOI:** 10.3390/molecules15117724

**Published:** 2010-10-29

**Authors:** Gökhan Kök, Tamer Karayıldırım, Kadir Ay, Emriye Ay

**Affiliations:** 1 Chemistry Department, Faculty of Science, Ege University, Bornova, İzmir 35100, Turkey; E-Mails: gokhan.kok@ege.edu.tr (G.K.); tamer.karayildirim@ege.edu.tr (T.K.); 2 Chemistry Department, Faculty of Arts and Sciences, Celal Bayar University, Campus of Muradiye, Manisa 45030, Turkey; E-Mail: kadir.ay@bayar.edu.tr (K.A.)

**Keywords:** chloralose, trichloroethylidene, dialdofuranose, Knoevenagel-Doebner reaction, sugar chain extension

## Abstract

The synthesis of new *α,β*-unsaturated furanuronic acid derivatives of *α*-*gluco*- (**3**), *β*-*gluco*- (**6**) and *β*-*manno*-chloraloses (**9**) via a convenient one pot procedure using the Knoevenagel-Doebner reaction approach are described. The dialdofuranose derivatives were reacted with malonic acid under Knoevenagel-Doebner reaction conditions and (*E*)-*α,β*-unsaturated furanuronic acid derivatives were obtained.

## 1. Introduction

In Organic Chemistry the well-known Knoevenagel reaction is defined by condensation of an aldehyde or ketone with compounds possessing active methylene groups such as malonic esters, or malononitrile to yield an *α,β*-unsaturated carboxylate products [1]. If the condensation reaction is set up in the presence of catalytic amounts of piperidine in a basic organic solvent such as pyridine, it is called the “*Doebner modification of Knoevenagel reaction*” or the “*Knoevenagel-Doebner reaction* or *condensation*” [2,3]. A *trans*- or *E*-*α,β*-unsaturated carboxylic acid is obtained as a decarboxylation product when malonic acid is heated in pyridine [2,3]. These type of condensation reactions also have been used in carbohydrate chemistry in order to extend sugar chains [4,5,6,7].

The Knoevenagel reaction can afford different types of compounds, depending on the acidic or basic catalysis employed. The condensation of unprotected reducing sugars with 1,3-dicarbonylic compounds under acidic conditions was reported in 1956 by Gonzalez [8]. The ZnCl_2_-catalyzed Knoevenagel reaction in alcoholic solvents has been used recently as the first step of the syntheses of a cytotoxic compound, of selective inhibitors of L-fucosidases of *E*- and *P*-selectin ligands, of glyco- and peptidomimetics, and of furan amino acid analogs of D- and L-serine. The so-called “*Garcia Gonzalez reaction*” was reinvestigated in order to improve yields using milder conditions such as CeCl_3_, InCl_3_, Sc(OTf)_3_, FeCl_3_, and scandium cation-exchanged montmorillonite [9].

The Knoevenagel condensation under basic conditions was first investigated with D-glucosamine chlorhydrate as the sugar. The reaction was extended to aldose and carbon nucleophiles such as barbituric acids and pentane-2,4-dione. When barbituric acids were used unprotected sugars gave *C*-glycosyl barbiturates. Reaction of pentoses or hexoses with Meldrum’s acid in DMF gave the unsaturated lactone. The condensation of pentane-2,4-dione with unprotected sugars in alkaline aqueous media was explored by the group of Lubineau. The condensation product of xylose with pentane-2,4-dione using NaOH, when reduced gave an activator of glycosaminoglycans biosynthesis used in cosmetic skincare products such as L’Oreal’s Pro-Xylane^TM^ [9].

The *aldehydo*-derivatives of monosaccharides readily react with malonic acid in pyridine in the presence of piperidine catalyst [4]. The Knoevenagel-Doebner reaction of open-chain and protected monosaccharides is known in the literature [4,10,11,12,13,14]. For instance, 2,3-dehydro-2,3-dideoxyaldonic acids of starting monosaccharides were obtained via Knoevenagel-Doebner condensation of free L-arabinose, D-xylose and D-galactose with malonic acid [10]. Furthermore, the condensation products of protected monosaccharides such as *aldehydo*-1,2;3,4-di-*O*-isopropylidene-*α*-D-*galacto*-1,6-*hexadialdo*-1,5-pyranose [11] and 2,4-*O*-ethylidene-D-erythrose and threose [12] were reported.

Knoevenagel-Doebner syntheses have also been performed with the monomethyl ester of malonic acid and unsaturated chain-extended monosaccharide products were thus obtained. When 2,3-*O*-iso-propylidene-D-glyceraldehyde and 2,3;4,5-di-*O*-isopropylidene-D-arabinose were used as a starting materials, methyl *trans*-2,3-dideoxy-4,5-*O*-isopropylidene-D-*glycero*-pent-2-enoate [13] and methyl *trans*-2,3-dideoxy-4,5;6,7-di-*O*-isopropylidene-D-*arabino*-hept-2-enoate [14] were formed, respectively, under very mild conditions.

Chloralose was firstly synthesized by Arthur Heffter in 1889 from the condensation of D-glucose and trichloroacetaldehyde (chloral) in the presence of an acid catalyst [15]. While two optical isomers were obtained *α*-glucochloralose (**1**) (*α*-chloralose) and *β*-glucochloralose (**4**) (*β*-chloralose) from the reaction of D-glucose with chloral, only *β*-isomers were obtained in the syntheses of *β*-xylochloralose [16] from D-xylose, *β*-arabinochloralose [17] from D-arabinose, *β*-galactochloralose [18] from D-galactose, *β*-mannochloralose (**7**) [19] from D-mannose via the same synthetic procedure.

Chloraloses are furanose-type cyclic acetals of pentoses and hexoses containing a 1,2-*O*-trichloro-ethylidene ring. Although, trichloroethylidene rings are highly stable under acidic and mildly basic conditions [19], they are unstable in strong bases such as a potassium *tert*-butoxide. The HCl elimination from trichloroethylidene ring of a chloralose with a strong base such as alkoxides gives a cyclic ketene acetal [17,19,20]. The trichloroethylidene group can be removed by hydrogenation with Raney nickel followed by acidic hydrolysis [21].

Trichloroethylidene acetals such as *α*-chloralose (**1**) [(*R*)-1,2-*O*-(2,2,2-trichloroethylidene)-*α*-D-glucofuranose] are potential biologically active compounds [22]. *α*-Chloralose was used in humans till the early part of the twentieth century [23]. Recently, it has been widely used as a bird repellent, a rodenticide, and in neuroscience and veterinary medicine as an anesthetic and sedative [22,23,24,25]. It has been characterized as a molecule which has potent *central nervous system* activity and evaluated in human and animal models for its therapeutic properties [26,27]. It is an anesthetic that exerts minimal cardiovascular depression, little change in blood pressure and no change or only a small reduction in heart rate, thus it is widely used in animals [28].

In addition to the well-known effects of chloraloses, arabinochloralose has been used as an intermediate compound for the development of new antituberculosis drugs in pharmaceutical research [29]. *Spiro*-endoperoxide chloraloses [30,31] and aminochloraloses [32] were synthesized and investigated antimicrobial activity against some microorganisms.

## 2. Results and Discussion

**Scheme 1:**
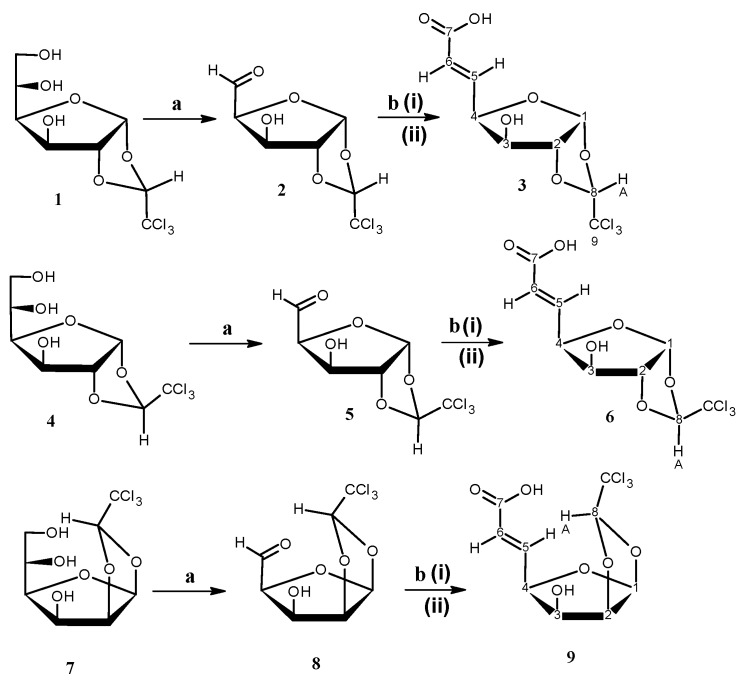
(a) NaIO_4_ in MeOH-H_2_O; (b) i. CH_2_(COOH)_2_ in piperidine cat. in pyridine at 60 ºC, ii. 6 N HCl.

*α,β*-Unsaturated carboxylic acid-containing sugar derivatives **3, 6,** and **9** were synthesized from compounds **1, 4,** and **7**, respectively, as shown in Scheme 1. The structure of the Knoevenagel-Doebner condensation products **3, 6,** and **9** was confirmed by using spectral methods (FTIR, ^1^H- and ^13^C-NMR). In the FT-IR spectrum of the new dialdofuranose compound **8**, the bands corresponding to the –OH and C=O are observed at 3,493 cm^-1^ and 1,737 cm^-1^, respectively. The aldehyde proton of this compound appeared as a singlet at 9.65 ppm in the ^1^H-NMR spectrum, and the carbonyl group peak appeared at 201.8 ppm in the ^13^C-NMR spectrum.

In the IR spectra of compounds **3, 6,** and **9** a broad OH absorption (stretching) band at 3,218, 3,453 and 3,446 cm^-1^, typical C=O absorption bands at 1,718, 1,702 and 1,693 cm^-1^ and characteristic C=C bands at 1,662, 1,668 and 1,658 cm^-1^ appeared, respectively. The –OH stretching bands of the carboxylic acid moieties were displayed at 3,481, 3,453 and 3,537 cm^-1^.

The ^1^H-NMR and ^13^C-NMR spectral data of compounds **3, 6,** and **9** are listed in Table 1. The hydroxyl and carboxylic acid group protons appeared as singlets at 5.48, 5.55, 5.76 ppm and 12.32, 12.42, 12.50 ppm, respectively. The large coupling constants (15.6 to 16.0 Hz) between 5-H and 6-H prove the formation of the *E*-isomer [33].

Long range couplings (1.2 to 2.0 Hz) were observed between H-4 and H-6 of the Doebner products. For compound **3**, this finding was previously explained as a result of a twisted conformation of the furanose ring causing the *endo*-trichloromethyl group to approach the 4-H hydrogen hence shifting it downfield [20,21].

The ^13^C-NMR spectra of compounds **3, 6,** and **9** are also consistent with the proposed structures, exhibiting two double bond carbon signals (123.3-141.9; 142.2-124.5; 143.7-123.8 ppm) and a carbonyl carbon signal (168.1, 167.2, 167.2 ppm) (Table 2**)**. The positive ion mode APCI-MS analysis of compound **3** gave molecular ion peaks at *m/z* 317/319/321 (18 %) (3 × chlorine isotopic pattern), 353/355/357/359 [[M + H]^+^ + Cl], 100 %, (4 × chlorine isotopic pattern), as the base peak groups.

A two pot alternative synthesis of similar compounds has been reported previously [27,31,33]. Hence, now we report synthesis of *α,β*-unsaturated furanuronic acid derivatives via a more facile one pot procedure using the Knoevenagel-Doebner reaction approach. This study is the first report of the synthesis of (*E*)-*α,β*-unsaturated furanuronic acid derivatives of *α*-*gluco*-, *β*-*gluco*- and *β*-*manno*- chloralose.

## 3. Experimental

### 3.1. General

Melting points were measured with a Gallenkamp electrotermal melting point apparatus in capillary tubes and are uncorrected. ^1^H-NMR (400 MHz) and ^13^C-NMR (100 MHz) spectra were recorded on a Varian AS 400 NMR spectrometer. APCI positive polarity (30 eV) mass spectra were recorded on Agilent 1100 (LC-MSD) mass spectrometer. IR spectra were recorded on Perkin Elmer Spectrum 100 FTIR Spectrometer. Optical rotation measurements were carried out on an Rudolph Research Analytical Autopol II digital polarimeter. TLC and column chromatography were performed on precoated aluminium plates (Merck 5554) and silica gel 60 (Merck 7734), respectively. All solvent removals were carried out under reduced pressure. Compound **1** was commercially available from Sigma-Aldrich Corp. and contained **4** as an impurity. Compound **4** is soluble in cold methanol, but **1** is not, and thus, impurities of **4** in the crude **1** were removed with cold methanol. Compound **4** was purchased from Sigma-Aldrich Corp. and purified via crystallization from boiling methanol. Syntheses of (*R*)-1,2-*O-*(2,2,2-trichloroethylidene)-*α*-D-*xylo*-1,4-furanodialdose (**2**) from **1**, (*S*)-1,2-*O-*(2,2,2-trichloroethylidene)-*α*-D-*xylo*-1,4-furanodialdose (**5**) from compound **4** were performed according to the literature via periodate oxidation reaction [31,33]. Compound **7** was synthesized from D-mannose and chloral according to the literature procedure [19].

*5,6-Dideoxy-(R)-1,2-O-(2,2,2-trichloroethylidene)-α-D-xylo-hept-5-(E)-eno-1,4-furanuronic acid* (**3**). A solution of compound **2** (7.00 g, 0.024 mol) in pyridine (50 mL) was mixed with malonic acid (12.48 g, 0.12 mol) and piperidine (0.5 mL). The solution was stirred at 60 ºC for 30 minutes and completion of the reaction was monitored with TLC (toluene/MeOH, 8:2). The reaction mixture was poured into a 1:1 H_2_O/HCl solution (200 mL). The acidic solution was extracted with CH_2_Cl_2_ (3 × 100 mL). Then the organic phase was dried with Na_2_SO_4_ and solvent was removed under reduced pressure. The residue was purified via column chromatography on silica gel using CH_2_Cl_2_:CH_3_OH (95:5) as an eluent. Compound **3** was obtained as a white solid after recrystallisation from CH_2_Cl_2_. (3.25 g, 42%); m.p. 150-151 ºC; ${\left[\alpha \right]}_{D}^{22}$: -77.9 (*c* 0.51 in CH_3_OH); IR cm^-1^ (KBr): 3,481 (-COOH), 3,218 (-OH), 1,718 (C=O), 1,662 (C=C); ^1^H-NMR δ (CDCl_3_): 7-H 12.32 (s, 1H), 5-H 6.75 (dd, 1H, *J_5,6_* = 15.6 Hz), 1-H 6.10 (d, 1H, *J*_1,2_ = 3.9 Hz), 6-H 5.98 (dd, 1H), OH 5.48 (s, 1H), H_A_ 5.43 (s, 1H), 2-H 4,68 (d, 1H), 3-H 4.23 (d, 1H, *J*_3,4_ = 3.1 Hz), 4-H 5.00 (ddd, 1H, *J*_4,5_ = 5.5 Hz); ^13^C-NMR δ (CDCl_3_): 168.1 (COOH), 141.9, 123.3 (5-C, 6-C), 107.8, 105.9 (1-C, CHCl_3_), 97.2 (CCl_3_), 87.8, 81.8, 75.4 (2-C, 3-C, 4-C); MS *m/z* 318 [M + H]^+^, 18 %, 317/319/321 (3 × chlorine isotopic pattern), 353/355/357/359 [[M + H]^+^ + Cl], 100 %, (4 x chlorine isotopic pattern). Anal. Calcd. for C_9_H_9_Cl_3_O_6_: C, 33.83; H, 2.84. Found: C, 33.92; H, 2.83.

*5,6-Dideoxy-(S)-1,2-O-(2,2,2-trichloroethylidene)-α-D-xylo-hept-5-(E)-eno-1,4-furanuronic acid* (**6**). Synthesized from **5** (1 g, 3.60 mmol) by the same procedure described for **3**. White solid, yield: 0.725 g (63%); m.p. 210-212 ºC (dec.); ${\left[\alpha \right]}_{D}^{26}$: -290.7 (*c* 0.86 in CH_3_OH); IR cm^-1^ (KBr): 3,453 (-OH and -COOH), 1,702 (C=O), 1,668 (C=C); ^1^H-NMR δ (DMSO-d6): 7-H 12.42 (bs, 1H), 5-H 6.75 (dd, 1H, *J*_4,5_ = 5.6 Hz, *J_5,6_* = 15.8 Hz), 1-H 6.28 (d, 1H, *J*_1,2_ = 3.6 Hz), 6-H 5.98 (dd, 1H, *J*_4,6_ = 1.2 Hz), H_A_ 5.85 (s, 1H), OH 5.55 (bs, 1H), 4-H 4.76 (m, 1H), 2-H 4,75 (d, 1H), 3-H 4.19 (bs, 1H); ^13^C-NMR δ (DMSO-d6): 167.2 (COOH), 143.8, 123.8 (5-C, 6-C), 109.4, 105.9 (1-C, CHCl_3_), 100.4 (CCl_3_), 82.3, 79.0, 75.9 (2-C, 3-C, 4-C); Anal. Calcd. for C_9_H_9_Cl_3_O_6_: C, 33.83; H, 2.84. Found: C, 33.07; H, 2.52.

*(R)-1,2-O-(2,2,2-trichloroethylidene)-β-D-lyxo-1,4-furanodialdose* (**8**). The 1,4-furanodialdose derivative were synthesized according to previous literature [19]. A solution of compound **7** (5.00 g, 16.15 mmol) in methanol (160 mL) was mixed with an aqueous solution of NaIO_4_ (4.02 g, 19.23 mmol). TLC analysis showed that the synthesis of (*R*)-1,2-*O*-(2,2,2-trichloroethylidene)-*β*-D-*lyxo*-1,4-furanodialdose was completed in 3.5 hours at room temperature. Reaction mixture was filtered, the filtrate was concentrated under reduced pressure and extracted with CH_2_Cl_2_ (4 × 150 mL). The organic phase was dried over anhydrous Na_2_SO_4_ and then evaporated to afford dialdofuranose **8** (3.55 g, 79 %) as a pure white amorphous powder, m.p. 115-117 ºC (dec.); ${\left[\alpha \right]}_{D}^{26}$: 0.11 (*c* 0.17 in CH_3_OH); IR cm^-1^ (KBr): 3,493 (-OH), 1,737 (C=O); ^1^H-NMR δ (DMSO-d6): 5-H 9.65 (s, 1H), 1-H 6.13 (d, 1H, *J*_1,2_ = 3.6 Hz), H_A_ 5.54 (s, 1H), OH 6.10 (bs, 1H), 2-H 4.81 (dd, 1H, *J*_2,3_ = 3.6 Hz), 3-H and 4-H 4.60 (m, 2H). ^13^C-NMR δ (DMSO-d6): 201.8 (CHO), 109.4, 106.6 (1-C, CHCl_3_), 100.1 (CCl_3_), 84.1, 82.2, 73.9 (2-C, 3-C, 4-C); Anal. Calcd. for C_7_H_7_Cl_3_O_5_: C, 30.30; H, 2.54. Found: C, 29.83; H, 2.27.

*5,6-Dideoxy-(R)-1,2-O-(2,2,2-trichloroethylidene)-β-D-lyxo-hept-5-(E)-eno-1,4-furanuronic acid* (**9**). Synthesized from **8** (1 g, 3.60 mmol) by the same procedure described for **3**. White solid, yield: 0.83 g (72 %); m.p. 195-197 ºC; ${\left[\alpha \right]}_{D}^{26}$: -171.6 (*c* 1.02 in CH_3_OH); IR cm^-1^ (KBr): 3,537 and 3,446 (-OH and -COOH), 1,693 (C=O), 1,658 (C=C); ^1^H-NMR δ (DMSO-d6): 7-H 12.50 (s, 1H), 5-H 6.75 (dd, 1H, *J*_4,5_ = 5.6 Hz, *J_5,6_* = 16.0 Hz), 1-H 6.14 (d, 1H, *J*_1,2_ = 3.6 Hz), 6-H 5.99 (dd, 1H, *J*_4,6_ = 1.6 Hz), H_A_ 5.92 (s, 1H), OH 5.76 (bs, 1H), 3-H 4.84 (dd, 1H, *J*_3,4_ = 4.4 Hz), 2-H 4,39 (dd, 1H, *J*_2,3_ = 3.6 Hz), 4-H 3.73 (m, 1H); ^13^C-NMR δ (DMSO-d6): 167.2 (COOH), 143.7, 123.8 (5-C, 6-C), 109.4, 105.6 (1-C, CHCl_3_), 100.4 (CCl_3_), 82.3, 79.0, 75.9 (2-C, 3-C, 4-C); Anal. Calcd. for C_9_H_9_Cl_3_O_6_: C, 33.83; H, 2.84. Found: C, 33.20; H, 2.54.

## 4. Conclusions

In the present work, the one-pot synthesis of three new potentially biological active furanuronic acid derivatives based on the 1,2-*O*-(2,2,2-trichloroethylidene) unit from via a Knoevenagel-Doebner reaction of the corresponding trichloroethylidene pentadialdofuranose was achieved. The spectroscopic data of the condensation products indicate the formation of only *E*-isomers of the carbon-carbon double bonds.

## Figures and Tables

**Table 1:** The ^1^H-NMR (400 MHz) chemical shifts (*δ* ppm) and *J*_H,H_ values (Hz) of Knoevenagel-Doebner condensation products of chloraloses.

Comp.	1-H	H_A_	2-H	3-H	4-H	5-H	6-H	7-H	OH
**3**	6.10 d, *J*_1,2_= 3.9 Hz	5.43 s	4.68 d	4.23 d, *J*_3,4_ = 3.1 Hz	5.00 ddd, *J*_4,5_ = 5.5 Hz, *J*_4,6_ = 2.0 Hz	6.75 dd, *J*_5,6_ = 15.6 Hz	5.98 dd	12.32 s	5.48 s
**6**	6.28 d, *J*_1,2_= 3.6 Hz	5.85 s	4.75 d	4.19 bs	4.76 m	6.75 dd, *J*_5,6_ = 15.8 Hz, *J*_4,5_ = 5.6 Hz	5.98 dd, *J*_4,6_ = 1.2 Hz	12.42 bs	5.55 bs
**9**	6.14 d, *J*_1,2_= 3.6 Hz	5.92 s	4.39 dd, *J*_2,3_ = 3.6 Hz	4.84 dd, *J*_3,4_ = 4.4 Hz	3.73 m	6.75 dd, *J*_4,5_ = 5.6 Hz, *J*_5,6_ = 16.0 Hz	5.99 dd, *J*_4,6_ = 1.6 Hz	12.50 s	5.76 bs

**Table 2:** The ^13^C-NMR (100 MHz) chemical shifts (*δ* ppm) of Knoevenagel-Doebner condensation products of chloraloses.

Comp.	COOH	5-C, 6-C	1-C, CHCCl_3_,	CCl_3_	2-C, 3-C, 4-C
**3**	168.1	141.9, 123.3	107.1, 105.9	97.2	87.8, 81.8, 75.4
**6**	167.2	142.2, 124.5	108.7, 106.6	100.4	88.2, 81.1, 75.7
**9**	167.2	143.7, 123.8	109.4, 105.6	100.4	82.3, 79.0, 75.9
